# Antibacterial activity and composition of essential oils from *Pelargonium graveolens* L'Her and *Vitex agnus-castus* L

**Published:** 2012-12

**Authors:** A Ghannadi, MR Bagherinejad, D Abedi, M Jalali, B Absalan, N Sadeghi

**Affiliations:** 1 Department of Pharmacognosy, School of Pharmacy, Isfahan University of Medical Sciences, Isfahan, Iran; 2 Department of Pharmaceutical Biotechnology, School of Pharmacy, Isfahan University of Medical Sciences, Isfahan, Iran; 3 Isfahan Pharmaceutical Sciences Research Center, Isfahan University of Medical Sciences, Isfahan, Iran; 4 Department of Nutrition, School of Health, Isfahan University of Medical Sciences, Isfahan 8174673461, Iran

**Keywords:** Antibacterial activity, GC-MS, Essential oils, *Pelargonium graveolens* L'Her, *Vitex agnus-castus* L

## Abstract

**Background and Objectives:**

Essential oils are volatile compounds that have been used since Middle Ages as antimicrobial, anti-inflammatory, sedative, local anesthetic and food flavoring agents.

In the current study, essential oils of *Pelargonium graveolens* L'Her and *Vitex agnus-castus L*. were analyzed for their antibacterial activities.

**Materials and Methods:**

The chemical compositions of essential oils were characterized by GC-MS. Disc diffusion method was used to study antimicrobial activity.

**Results and Conclusion:**

Inhibition zones showed that the essential oils of the two plants were active against all of the studied bacteria (except *Listeria monocytogenes*). The susceptibility of the strains changed with the dilution of essential oils in DMSO. The pure essential oils showed the most extensive inhibition zones and they were very effective antimicrobial compounds compared to chloramphenicol and amoxicillin. The most susceptible strain against these two essential oils was *Staphylococcus aureus*. It seems that *β*-citronellol is a prominent part of *P. graveolens* volatile oil and caryophyllene oxide is a famous and important part of *V. agnus-castus* volatile oil and their probable synergistic effect with other constituents are responsible for the antibacterial effects of these oils. However further studies must be performed to confirm the safety of these oils for use as antimicrobial agents and natural preservatives in different products.

## INTRODUCTION

Essential oils are volatile, natural, complex compounds that are produced by plants as secondary metabolites for protection against bacteria, viruses, fungi and pests (1). They also have an important role in dispersion of pollens and seeds by attracting some insects. In Middle Ages essential oils were used for preservation of foods and as flavoring, antimicrobial, analgesic, sedative, anti-inflammatory, spasmolytic and locally anesthetic remedies (2–4). But characterizing these properties in laboratory dated back to the early 1900s. At present, about 3000 essential oils are known and 300 of them are used commercially in different industries such as pharmaceutical, agronomic, food, sanitary, cosmetic and perfume (5). Today, antioxidant, antitumor and antiviral, antifungal and antibacterial activity of essential oils and their constituents is widely studied (6–14). In the other hand, Antimicrobial resistance (AMR) is now a global concern. Resistant organisms such as bacteria, viruses and some parasites are able to live in the presence of antimicrobial medicines, such as antibiotics, antivirals, and antimalarials, so that standard treatments become ineffective and infections can spread to others (15). AMR is the result of misuse of antimicrobial medicines and develops when a microorganism mutates or acquires a resistance gene. So finding new antimicrobial agent is one of the fields that many researchers are interested in. Among these studies may be the most focus is on natural antimicrobial agent such as essential oils.

The *Pelargonium* (Geraniaceae family) and *Vitex* (Verbenaceae family) genera are two important sources of foods, medicines and cosmetics in the world and Iran. They are also sources of distilled volatile oils (16–20). These genera have been found to possess significant pharmacological and biological activities, including antioxidant, anti-neuroinflammatory, anti-influenza, anticancer, antimicrobial and antifungal activity (16, 17, 20–23).

*Pelargonium graveolens* L'Her and *Vitex agnus-castus* L. are the most important species of their genera and used in several foods, remedies and cosmeceuticals (16–18, 20, 21, 24).

*P. graveolens* aerial parts are used in folk medicine of Iran and the world as a food and tea drinks additive and for relieving of some gastrointestinal, topical, dental and cardiovascular disorders (17, 18, 20, 24). There are some reports on the phytochemical analysis of the plant found in the literature. Some scientific studies on *P. graveolens* showed the presence of constituents belonging mainly to the groups of essential oils, phenolics and flavonoid (17, 20–22, 25–28). The flavor composition in *Pelargonium* has been studied by various authors (20, 22, 25–28).

*V. agnus-castus* fruits have been used in food as flavor and spice component and in Iranian traditional and folk medicine as a hormone-like remedy for relieving of menstrual problems and as a mild tranquilizer and digestive (16, 18). Phytochemical studies on *V. agnus-castus* revealed volatile oil, flavonoids, coumarins, terpenes were the main components of the plant (16, 23, 24, 29). Volatile oil constituents of those herbs are responsible for their major pharmacological activities (16, 17, 21).

Although the chemical compositions of the volatile oils of these plants are well studied, to our best knowledge no research has so far been conducted on these Persian sources and their antibacterial effects. The goal of this investigation was to determine whether the volatile oils of *P. graveolens* and *V. agnus-castus* possess antibacterial activities against some common pathogen and food-borne bacterial pathogens.

## MATERIALS AND METHODS

### Plant materials

The aerial parts of *P. graveolens* were collected during the flowering period from cultivated plants around Kashan in Isfahan province, centre of Iran in June 2001 and the seeds of *V. agnus-castus* had been collected from Mazandaran province, north of Iran in July 2001. The species were identified in herbarium department of Science and Research Branch, Tehran Islamic Azad University, Tehran, Iran by Dr. Iraj Mehregan. Voucher specimens of the plants were deposited at the herbarium department in our school.

### Volatile oils isolation

The powdered air-dried plant parts were subjected to hydro-distillation in a Clevenger-type apparatus separately until there was no significant increase in the volume of the oil collected for 3 h. The volatile oils were dried over anhydrous sodium sulfate and stored at 4 °C in the dark for analysis and further antibacterial studies (30, 31).

### GC-MS

GC/MS analysis was performed on a Hewlett Packard 5972A mass selective detector coupled with a Hewlett Packard 6890 gas chromatograph, equipped with a cross-linked 5% PH ME siloxane HP-5MS capillary column (30m×0.25 mm, film thickness 0.25 µm).

The GC operating conditions were as follows: carrier gas, helium with a flow rate of 2 mL/min; column temperature, 60°-275°C at 4°C/min; injector and detector temperatures, 280°C; volume injected, 0.1 µL of the oil; split ratio, 1:25.

The MS operating parameters were as follows: ionization potential, 70 ev; ion source temperature, 200°C; resolution, 1000.

Identification of components in the oil was based on GC retention indices relative to *n*-alkanes and computer matching with the Wiley 275.L library, as well as by comparison of the fragmentation patterns of the mass spectra with those reported in the literature (32–35).

### Microorganisms

The antibacterial activity of essential oils was investigated against six bacterial species. Test organisms included *Listeria monocytogenes* (PTCC 1297), *Salmonella enteritidis* (PTCC 1091), *Pseudomonas aeruginosa* (PTCC 1074), *Escherichia coli* (PTCC 1330), *Staphylococcus aureus* (PTCC 1112) and *Bacillus subtilis* (PTCC 1023). Lyophilized microorganisms (MOs) were sub-cultured in Muller-Hinton broth (MHB, Oxoid, UK) then plated onto Muller-Hinton agar (MHA, Oxoid, UK) as working cultures. The bacterial suspension was prepared in normal saline by picking colonies from an overnight plate. Also, all cultures were maintained at -70 °C in 20% glycerol for further analysis.

### Antibacterial activity assay

The antimicrobial effect of essential oils is usually determined by an initial in vitro screening method such as disc diffusion method where a filter disc is impregnated with the essential oils and placed on the surface of inoculated agar plates. In current study this method was also applied. Muller-Hinton agar plates were prepared according to bi-layer method. The base layer was 30 ml of MHA and the seed layer was consisted of 9 ml MHA and 1 ml microbial suspension (10^8^cfu/ml) to achieve final concentration of 10^7^cfu/ml. The mixture of seed layer was uniformly spread on base layer. The concentration of microbial suspension was determined spectrophotometrically (580 nm, 0.2 OD).

Sterile paper discs (6.4 mm diameter, Padtan, Iran) were impregnated with 15 µl of neat essential oils and two different concentrations (1/2, 1/4 prepared by dilution in DMSO). The discs were dried after incubation at 37 °C for 24 h. Every six discs were placed on a MHA plates surfaces. Negative controls were prepared using 15 µl of DMSO. Amoxicillin (25µg/disc) and chloramphenicol (30µg/disc) were used as positive control to determine the sensitivity of microbial strains and DMSO as negative control. After pre-incubation (60 min at 4°C), inoculated plates were incubated at 37 °C for 24 h. Antimicrobial activity was evaluated by measuring the inhibition zone on the surface of plates and the results were reported as Mean±SD after three repeats.

## RESULTS

The volatile oils obtained after hydro-distillation of the aerial parts of *P. graveolens* and the seeds of *V. agnus-castus* gave average yields of 0.8% and 1% (w/v), based on dry weights. The results obtained for the qualitative and quantitative analyses of the oils can be seen in Tables 1 and 2. More than twenty compounds were identified in each volatile oil, accounting for 94.3% and 97.5% of the *Pelargonium* and *Vitex* oils.

**Table 1:** Composition (%) and Retention Indices (RI) of the Essential Oil of *Pelargonium graveolens* L'Her

No	RI^a^	Compound	%^b^
1	935	*α*-pinene	1.0
2	1095	linalool	5.1
3	1107	*cis*-rose oxide	2.8
4	1124	*trans*-rose oxide	1.1
5	1151	*para*-menthone	2.7
6	1162	isomenthone	7.3
7	1186	*α*-terpineol	0.4
8	1227	*β*-citronellol	36.4
9	1239	neral	0.4
10	1255	geraniol	10.7
11	1275	citronellyl formate	12.1
12	1301	geranyl formate	2.6
13	1356	citronellyl acetate	0.6
14	1378	*α*-cofaene	0.5
15	1385	geranyl acetate	1.5
16	1421	*β*-caryophyllene	3.4
17	1446	citronellyl propanoate	1.0
18	1457	*α*-humulene	0.8
19	1483	germacrene-D	1.3
20	1528	*δ*-cadinene	0.9
21	1534	citronellyl *n*-butyrate	1.1
22	1568	geranyl *n*-butyrate	1.3
23	1587	caryophyllene oxide	1.2
24	1710	gerany ltiglate	1.0

a Retention indices on HP-5MS capillary column

b %: Calculated from TIC data

**Table 2:** Composition (%) and Retention Indices (RI) of the Essential Oil of *Vitex agnus-castus* L.

No	RI^a^	Compound	%^b^
1	954	camphene	0.2
2	1145	camphor	1.1
3	1175	4-terpineol	0.7
4	1191	*α*-terpineol	2.6
5	1297	*n*-tridecane	0.3
6	1348	α-terpinyl acetate	11.6
7	1351	citronellyl acetate	0.8
8	1398	*n*-tetradecane	3.3
9	1417	*β*-caryophyllene	5.8
10	1430	*β*-gurjunene	1.2
11	1436	trans-*α*-bergamotene	1.9
12	1441	*β*-humulene	0.8
13	1445	*trans*- *β*-farnesene	4.8
14	1456	*α*-humulene	2.7
15	1495	bicyclogermacrene	8.4
16	1500	*n*-pentadecane	2.6
17	1523	*γ*-cadinene	0.5
18	1578	spathulenol	4.7
19	1584	caryophyllene oxide	24.9
20	1602	*n*-hexadecane	12.5
21	1708	*n*-heptadecane	4.4
22	1811	*n*-octadecane	1.7

a Retention indices on HP-5MS capillary column

b %: Calculated from TIC data

Growth inhibition zones produced by essential oil of *P. graveolens* are reported in Table 3.

**Table 3:** Growth inhibition effect of *Pelargonium graveolens* essential oil (zone size, mm).

*Microorganisms*	*Essential oil Concentrations (Diluted in DMSO)*			*Standard Antibiotic*	

	Neat	1/2	¼	Amoxicillin (25 µg/disc)	Chloramphenicol (30 µg/disc)
*E. coli*	9.5±0.7	8.1±0.3	7.0±0.0	16.3±0.1	13.6±0.3
*P. aeruginosa*	25.3±1.1	17.3±1.1	15.3±1.1	27.1±0.2	19.2±0.2
*S. aureus*	42.0±3.2	34.0±2.8	14.3±1.5	39.5±0.1	28.7±0.3
*B. subtilis*	16.5±0.7	13.5±0.7	11.0±0.0	23.0±0.1	16.8±0.1
*S. enteritidis*	9.0±0.0	NE*	NE	16.1±0.3	14.6±0.6
*L. monocytogenes*	NE	NE	NE	35.0±0.1	16.0±0.2

* No Effect

Table 4 summarized the antibacterial activity of *V. agnus–castus* volatile oil. This oil had no effect on *L. monocytogenes* and *E.coli* but other four strains were susceptible and *S. aureus* was again the most sensitive one.

**Table 4:** Growth inhibition effect of *Vitex agnus-castus* essential oil (zone size, mm).

Microorganisms	Essential oil Concentrations (Diluted in DMSO)			Standard Antibiotic	

	Neat	1/2	¼	Amoxicillin (25 µg/disc)	Chloramphenicol (30 µg/disc)
*E. coli*	NE*	NE	NE	16.3±0.1	13.6±0.3	
*P. aeruginosa*	41.0±0.7	31.0±3.4	27.0±5.6	27.1±0.2	19.2±0.2	
*S. aureus*	50.0±0.0	42.8±3.2	22.3±3.2	39.5±0.1	28.7±0.3	
*B. subtilis*	11.0±0.7	8.5±0.7	NE	23.0±0.1	16.8±0.1	
*S. enteritidis*	9.0±0.0	NE	NE	16.1±0.3	14.6±0.6	
*L. monocytogenes*	NE	NE	NE	35.0±0.1	16.0±0.2	

* No Effect

## DISCUSSION

The main constituents of *P. graveolens* volatile oil were *β*-citronellol (36.4%) and citronellyl formate (12.1%). Caryophylle oxide (24.9%), *n*-hexadecane (12.5%) and α-terpenyl acetate (11.6%) were the main constituents of the volatile oil of *V. agnus-castus*. There are variations in the composition of these volatile oils in comparison to the volatile oils of other countries. These differences seem to depend on climate changes and conditions and types and methods of distillation (16, 17, 20, 22, 23, 26–29).

It seems that the essential oils of *P. graveolens* and *V. agnus-castus* cultivated in Iran were characterized as citronellol and caryophyllene oxide chemotypes, respectively. All bacterial strains were susceptible against *P. graveolens* essential oil except *L. monocytogenes*. The most sensitive strain was *S. aureus* and this essential oil had a good inhibiting effect on its growth. *V. agnus-castus* oil had no effect on *L. monocytogenes* and *E. coli* but other four strains were susceptible and *S. aureus* was again the most sensitive one.

All strains were also sensitive to the control antibiotics (amoxicillin and chloramphenicol). The essential oils of the two plants were active against all of the bacteria (except *L. monocytogenes*) and the susceptibility of the strains changed with the dilution of essential oils in DMSO. The pure and neat essential oils showed the most extensive inhibition zones and they were very effective antimicrobial system comparing to chloramphenicol and amoxicillin. The activity of the oils would be expected to relate to the composition of the plant essential oils and possible synergistic interaction between components. High proportions of *β*-citronellol and caryophyllene oxide in our oils make them interesting and valuable subjects for food, medicine, aromatherapy and cosmetics industries where an antiseptic, clean and fresh characteristics flavor and fragrance is desired. The food protective and antimicrobial properties of *β*-citronellol as prominent part of *P. graveolens* volatile oil (36) and antifungal, analgesic and anti-inflammatory activities of caryophyllene oxide as famous and important part of *V. agnus-castus* volatile oil (37, 38) can be considered and may be suitable for use in further nutraceutical and pharmaceutical vehicles.

The antibacterial effects of the essential oils of different species of *Pelargonium* and *Vitex* including *P. tomentosum, P. denticulatum, P. odoratissimum, P. fragrans and V. negundo and V. trifolia* were evaluated previously. They were found to give promising results against several bacteria (39, 40, 41). There are similar compounds in both of our essential oils. They are α-terpineol, citronellyl acetate, β-caryophyllene, α-humulene and caryophyllene oxide. It seems part of the antibacterial effects of these two essential oils are related to these similar compounds especially β-caryophyllene and caryophyllene oxide (38, 42, 43).

The observed antibacterial properties show that they have a good potential for use as antimicrobial agents and natural preservatives in different products. However, further studies are needed to explore the efficiency of suitable concentration and to examine how the combined product exhibit antimicrobial activity. More studies should also confirm the safety of these essential oils and introduce the main compound responsible for antibacterial activity.
